# Study on the Context-Aware Middleware for Ubiquitous Greenhouses Using Wireless Sensor Networks

**DOI:** 10.3390/s110504539

**Published:** 2011-04-27

**Authors:** Jeonghwang Hwang, Hyun Yoe

**Affiliations:** School of Information and Communication Engineering, Sunchon National University, Maegok-dong, Suncheon-si, Jeollanam-do 540-742, Korea; E-Mail: jhwang@sunchon.ac.kr

**Keywords:** WSN, ubiquitous society, agriculture, context-aware, middleware, greenhouse

## Abstract

Wireless Sensor Network (WSN) technology is one of the important technologies to implement the ubiquitous society, and it could increase productivity of agricultural and livestock products, and secure transparency of distribution channels if such a WSN technology were successfully applied to the agricultural sector. Middleware, which can connect WSN hardware, applications, and enterprise systems, is required to construct ubiquitous agriculture environment combining WSN technology with agricultural sector applications, but there have been insufficient studies in the field of WSN middleware in the agricultural environment, compared to other industries. This paper proposes a context-aware middleware to efficiently process data collected from ubiquitous greenhouses by applying WSN technology and used to implement combined services through organic connectivity of data. The proposed middleware abstracts heterogeneous sensor nodes to integrate different forms of data, and provides intelligent context-aware, event service, and filtering functions to maximize operability and scalability of the middleware. To evaluate the performance of the middleware, an integrated management system for ubiquitous greenhouses was implemented by applying the proposed middleware to an existing greenhouse, and it was tested by measuring the level of load through CPU usage and the response time for users’ requests when the system is working.

## 1. Introduction

Wireless Sensor Network (WSN) technology is one of the important technologies for implementing a ubiquitous society, and it is applied into various fields such as distribution, logistics, construction, transportation, agriculture, defense, medicine, *etc.* [1], and in particular, it can be applied to the agricultural environment, production management, distribution, *etc.* to increase the productivity of agricultural and livestock products and secure transparency of distribution channels in the agricultural sector [2,3].

Ubiquitous agriculture (u-Agriculture) is defined as a technology that increases agricultural added value and productivity by combining ubiquitous technologies such as WSNs with agricultural sector applications, and recently, various pilot projects and research projects involving the design and implementation of monitoring systems combining WSN technologies have been tried in various of agricultural and livestock production environments for product management and distribution in applications such as greenhouses and livestock barns, *etc.* [4–8].

In order to easily construct such a u-Agriculture environment, middleware is required to connect WSN hardware, applications, and enterprise systems [9,10]. It is said that middleware is a technology to filter a large volume of data collected from many heterogeneous WSNs, process event data, and then convert it into meaningful information, and furthermore, to transmit and process more efficiently a large number of contexts and data produced in a ubiquitous environment [11–16].

Recently, even though WSN middleware has been studied in various fields, there have been insufficient studies on middleware focused on application services in an agricultural environment where compared to other industries, IT technology has been inadequately applied [17]. Therefore, this paper would like to propose a context-aware middleware to efficiently process data collected from greenhouses by applying WSN technologies and to implement combined services through organic data connectivities.

In greenhouses using WSN technologies, soil sensor and environmental sensor nodes are installed inside/outside the greenhouse in order to collect environmental information for monitoring the greenhouse crop growth, and these sensor nodes constitute a wireless sensor network to collect environmental and soil information in the greenhouse.

In order to provide various services to users by using wireless sensor networks composed of heterogeneous sensors, WSN middleware should be capable of converting collected sensor data into a common form, reducing server loads by using some data filtering function(s), and providing intelligent context-aware and event service functions.

The proposed context-aware middleware could maximize scalability and usability of the system by abstracting heterogeneous sensor nodes installed for collecting greenhouse environmental information, enabling data filtering, event processing and context-aware processing, and integrating different forms of data through this.

The proposed context-aware middleware was applied to a ubiquitous greenhouse integrated management system, and we could thus maximize scalability and operability of the system and improve the productivity of greenhouse crops and user convenience.

This paper is organized as follows: Section 2 describes related studies, Section 3 explains design of the proposed context-aware middleware, Section 4 implements the proposed middleware and evaluates its performance through applied examples, and finally, Section 5 concludes this paper by describing the conclusions and the future study topics.

## 2. Related Research

### 2.1. Wireless Sensor Networks Middleware

WSN middleware is a software layer that exists physically between hardware such as sensor nodes, gateways, *etc.* and applications, that supports the flexible integration of hardware and applications. In addition, it could be defined as a software to help provide services such as distributed computing environments, remote procedure calls, messaging to users, regardless of the hardware, operating system, network, *etc*. used. The recently studied WSN middleware can be divided according to its location into in-network schemes to be installed at sensor nodes, server-side schemes installed at servers, and hybrid schemes combining two schemes [10].

#### 2.1.1. In-Network Schemes

Typical WSN in-network scheme middlewares, in which the middleware is installed at sensor nodes, are Mate developed at UC Berkeley, Impala developed at Princeton University, and Agilla developed at Washington University.

Mate is a virtual machine based middleware developed for sensor networks. It works on sensor nodes by installing TinyOS, is equipped with its own byte code interpreter, and supports a mechanism to distribute new codes through a contagion model. The high-level interface of Mate makes a complex program very simple, and it minimizes the resources required to send a new execution module to sensor nodes [18].

Impala was begun as part of the ZebraNet project at Princeton University. ZebraNet is a project to study the movement and breeding of animals such as zebras using sensor network technologies. Impala focuses on modularization, adaptability, and restoration of applications; software updated in Impala is delivered to each sensor node via wireless networks, and each node could carry out updates under the condition that the system is working. In addition, it improves performance of software systems, energy efficiency, stability, and provides dynamic applied adaptability for various parameters and device failures, *etc*. [19].

Agilla is a mobile agent-based middleware to allow codes and states of sensor nodes to move using WSNs. Agilla controls ensure the flexible propagation of sensor node state information. Agilla works on TinyOS, and carries out agent’s function at each node. Agilla provides a neighbor list and double space resources at each node. The neighbor list that includes addresses of neighboring nodes is used to move and copy information of each node. The double space provides a decoupled-mode for communication between agents [20].

#### 2.1.2. Server-Side Schemes

Typical server-side WSN middleware schemes, in which the middleware is installed on servers, are Cougar developed at Cornell University, SINA developed at the University of Delaware, and MiLAN developed at the University of Rochester.

Cougar is a distributed data processing system for sensor networks studied by the database research team at Cornell University. Even though most sensor network applications recently studied employ a scheme in which a base node collects all the data and data is processed at the center, Cougar carries out all the data access and processing in a distributed form. Cougar uses declarative queries so that it also has a property that users are hidden from the physical properties of the networks. It was designed as a system that could adapt dynamically to network variation, has high flexibility and scalability, and has fault tolerance [21].

SINA considers sensor networks as a distributed database to access sensor network information using a query form. SINA includes not only a method to restrict re-transmission of similar information from sensor nodes distributed at geographically close locations but also a lower level mechanism to perform hierarchical grouping of sensors for efficient data fusion [22].

MiLAN is middleware for sensor networks that was developed at the University of Rochester for health management tasks at smart medical homes. It computes a measurement plan to minimize total energy consumption to satisfy the reliability demands of various types of medical data desired by users, activates the corresponding sensors according to the plan, and provides services to collect measured data and deliver it to users [23].

#### 2.1.3. Hybrid Schemes

Hybrid scheme WSN middleware combines two of the schemes mentioned earlier; typical WSN middleware of this type are COSMOS developed at ETRI, DSWare developed at Virginia University, and TinyDB developed at UC Berkeley.

COSMOS, developed at ETRI, extracts core functions of middleware commonly required in various types of WSN application services, and brings about technology development and standardization for providing them as a standardized scheme. Major functions of COSMOS are supporting various types of queries involving massive simultaneous query processing for a large volume of sensor network environments, and supporting an abstraction function for heterogeneous sensor networks [24].

DSWare carries out data service functions by providing combined events defined by a certain pattern as a basic programming function, and has a feature of variously providing also real-time data service such as definition of deadlines for event reports and intervals for certain events, *etc.* [25].

TinyDB is middleware studied at UC Berkeley. It is a query system to obtain information from sensor networks driven on TinyOS. In other words, TinyDB collects data from motes installed in the environment to filter and arranges it to for sending to outside PCs. TinyDB considers sensor networks as a virtual distributed database, and supports SQL-like query language and SRT. In addition, TinyDB provides a simple sensor API for making PC applications that could extract data from sensor networks through queries, and a query generator in GUI form and a program to display results that also uses this API. To use TinyDB, TinyDB components based on TinyOS should be installed on each sensor node in the sensor network [26].

### 2.2. Comparison with Other Middlewares

Xiong and his colleagues have proposed an optimization of existing methods, called tuning adaptive margin failure detector (TAM FD), which significantly improves quality of service (QoS), especially in the aggressive range and when the network is unstable [14]. In addition, he and his colleagues have proposed a novel and efficient distributed flow control scheme for multirate multicast (MR-M), based on the well known Proportional Integral and Derivative (PID) controllers [15].

Zhou and his colleagues have developed context-aware middleware for multimedia services in heterogeneous networks. This context-aware middleware system facilitates diverse multimedia services in such heterogeneous network environments by combining an adaptive service provisioning middleware framework with a context-aware multimedia middleware framework [16]. In addition, he and and his colleagues have developed fully distributed scheduling schemes with the goal of minimizing the video distortion and achieving certain fairness [17].

In this paper, we have studied context-aware middleware applied to the particular environment of a WSN based greenhouse. The proposed middleware has a different system structure from existing middlewares, and uses an ontology model for representation of context because ontology could define some information between concept and relationship, and handle new contexts easily by using rule-based reasoning functions.

## 3. Design of the Proposed Context-Aware Middleware for a Ubiquitous Greenhouse

### 3.1. Ubiquitous Greenhouse

#### 3.1.1. System Architecture

The ubiquitous greenhouse (u-Greenhouse) is a system in which ubiquitous technology is applied to a greenhouse to control the environmental monitoring and control facilities of the greenhouse. The u-Greenhouse of this paper applies a WSN, which is a ubiquitous technology, as a basis system of the greenhouse [27].

In the u-Greenhouse of this paper, soil sensors and environmental sensors are installed inside/outside the greenhouse in order to collect environmental data relevant to the greenhouse’s crop growth such as illuminance, temperature, humidity, CO_2_ level, *etc.* and soil information like soil humidity, soil temperature, *etc.*, and these sensors together constitute a wireless sensor network to collect environmental and soil information from the greenhouse. In addition, CCTVs are installed inside/outside the greenhouse to collect real time image information to provide additional greenhouse and crop image information and preventing dangers such as burglary and fire.

**Figure 1.**
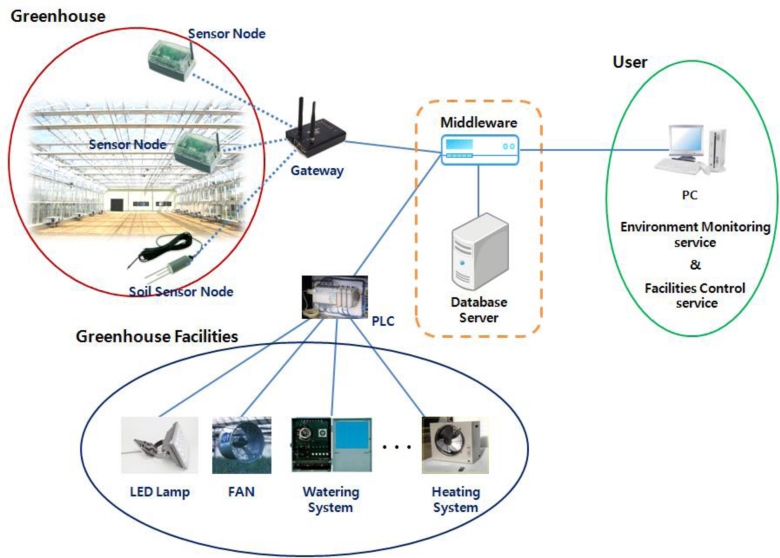
System structure of a ubiquitous greenhouse.

Such collected environmental and image information is stored in a server via the gateway, provided to users in real time through various interfaces, and environmental control facilities in the greenhouse can be automatically or manually controlled to ensure an optimum growth environment for the cultivated crops based on the collected information. Figure 1 shows the system structure of the ubiquitous greenhouse.

#### 3.1.2. System Components

The u-Greenhouse has installed ZigbeX 2.0 sensor nodes from Hanback Electronics and developed sensor nodes inside/outside the greenhouse in order to measure environmental information such as temperature, humidity, illuminance *etc.* in the greenhouse.

**Figure 2.**
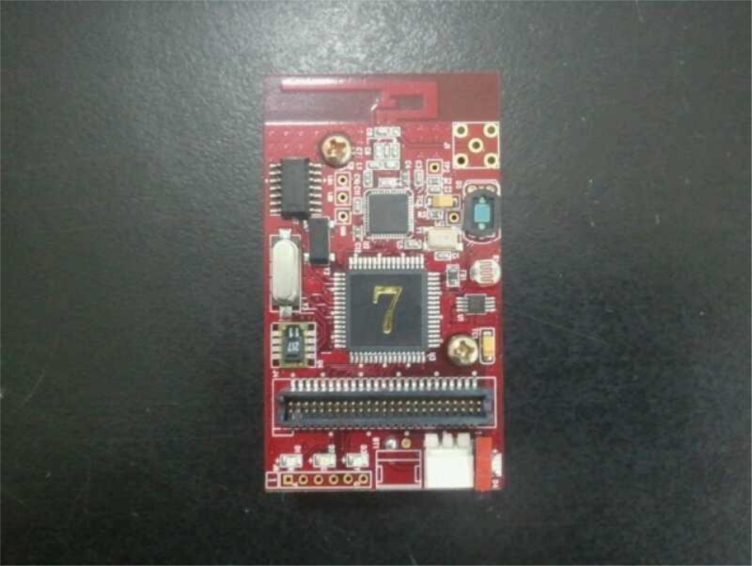
ZigbeX 2.0 sensor node from Hanback Electronics.

Figure 2 shows a ZigbeX 2.0 sensor node from Hanback Electronics. It is equipped with Atmel’s ATmega128L with 128 KB internal programmable RISC architecture as a MCU, and Chipcon’s CC2420 is used as a RF Transceiver. This ZigbeX unit outputs digital temperature/humidity data through a 14-bit ADC containing SHT11 temperature/humidity sensor as a multi-sensor device, and includes a Cds illuminance sensor with the maximum sensitivity at 540 nm. For power it uses two rechargeable AA size batteries of between 2.7∼3.6 VDC (1.2 rechargeable battery/1.5 Alkaline). The antenna for the RF Transmitter/Receiver is an F-type, which basically uses a PCB antenna with outdoor/indoor radius of 75 m∼100 m/20 m∼30 m, and a dipole antenna could be used as an option by users [28].

**Figure 3.**
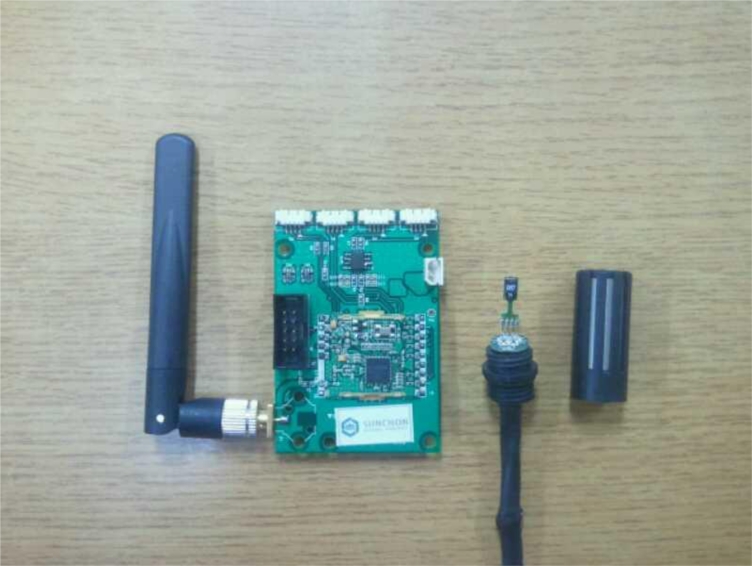
Environmental sensor node.

Figure 3 is a developed sensor node, which plays the role of receiving sensor data from the temperature/humidity sensor, processing data in the MSP430 MCU to send it to a relay node and a gateway via a CC2420 RF chip. In addition, the node is separated from the sensor in order to reduce the effects of any heat generated by the node on the sensor [29].

The MSP430 used in the sensor node is a 16-bit RISC, which has 48 Kbyte of program memory and 10 Kbyte RAM inside [30], so that it can process sensor data at high speed, and the CC2420 is a RF chip supporting ZigBee, which in turn supports 2,400∼2,483.5 MHz band, works as a DDDS scheme, and supports O-QPSK modulation scheme and has a 250 Kbps data rate so that low-power real-time wireless communication is possible [31].

SHT71 is used as an integrated temperature/humidity sensor. Operating power (2.4 V∼5.5 V) is relatively low and power consumption is also low, with an average value of 28 μA. It has a compensating memory, 14-bit A/D converter, digital 2-wire interface inside, and it can measure temperatures from −40 to 120 °C and has an accuracy error of ±0.5 °C. In addition, humidity is measured between 0 and 100%, with an accuracy error of 3.5%. 3.3 V of operating voltage is connected to the sensor node, and digital 2-wire is connected with circuits of MSP430 to process the greenhouse temperature and humidity information [32].

**Figure 4.**
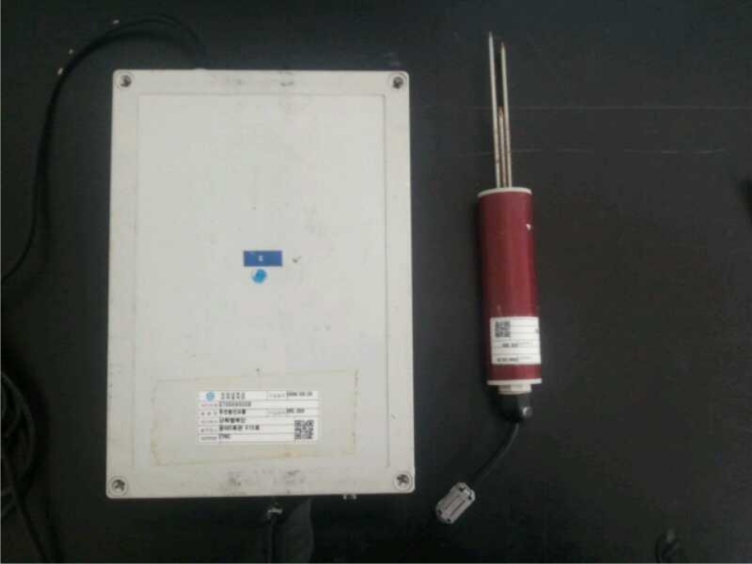
Soil sensor node.

Figure 4 shows a sensor node used to measure soil information such as soil temperature, soil humidity, *etc.* in the greenhouse. The soil sensor can store data by date using a downloading program without a data logger when connected with a computer via a relay. It can measure soil moisture from 0 to 99.9%, and the error rate is ±3%. Soil temperature can be measured from 0 to 60 °C, and the error rate is ±0.5 °C, so that its performance is excellent.

**Figure 5.**
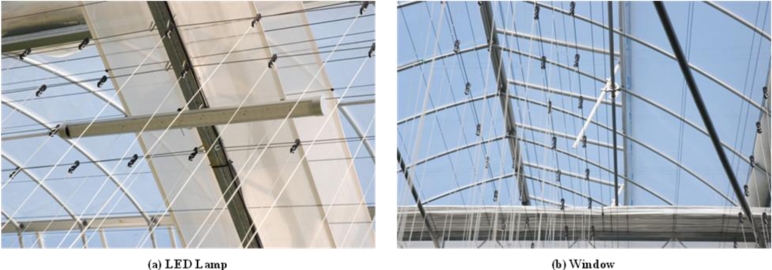

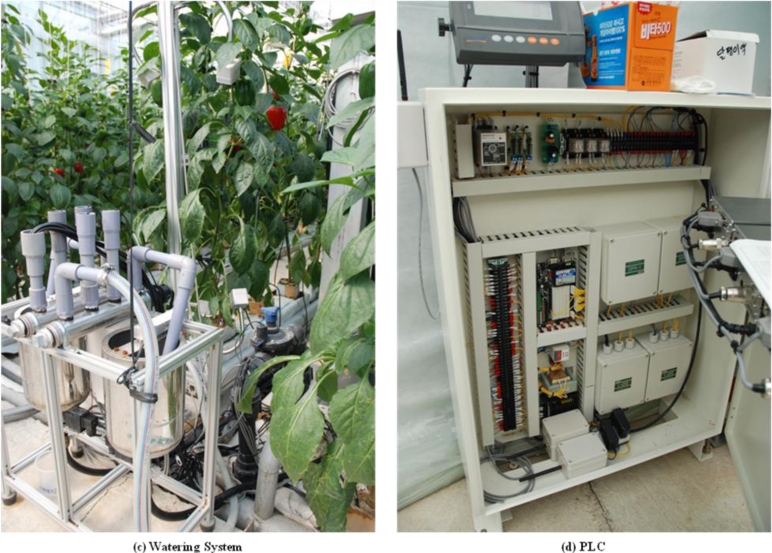
Environment control facilities and PLC.

To control greenhouse environment parameters influencing crop growth such as illuminance, temperature, humidity, CO_2_, *etc.* based on information collected from the environmental sensors and the soil sensor mentioned above, environment control facilities such as a ventilation and heating system, heat insulation system for reducing energy, shading curtain system following external brightness, circulating fan system regulating air flow in the facility, temperature control system of hot water/heating water, and artificial light source control system following external brightness and biometric information *etc.* are constructed in the greenhouse, and each environment control facility is controlled through a PLC. Figure 5 shows the environment control facilities and the PLC installed in the greenhouse.

### 3.2. Design of the Proposed Context-Aware Middleware

The context aware u-Greenhouse middleware is designed aimed at a system to smoothly interconnect two layers between the wireless sensor network basically composed of heterogeneous sensors and the system to provide various services to users, and to process data to effectively provide intelligent service functions that could recognize contexts based on sensor data.

#### 3.2.1. Requirements Analysis

Requirements for the middleware analyzed in this paper could be divided into three parts: sensor network interface, data process, and application service interface, according to the location of each function. The sensor interface carries out data abstracting functions, and the data process provides data filtering, various query processing functions for sensor data, and real-time management functions for sensor information. In addition, the application service interface supports connections with the outside through query processing, context aware services, and event services.

The data abstracting function solves the dependency of sensor nodes by recognizing sensors and converting collected sensor data into a common form after registering heterogeneous sensor nodes, which are ported on each other’s platform, to the middleware in advance.

The data filtering function is to avoid storing duplicated data to reduce the server’s load for data sent in real time, and the context aware service is a function that specifies numerical values for certain data and decides contexts intelligently, to autonomically control greenhouse facilities appropriately for an optimum crop growth environment in the greenhouse. The event service prevents unexpected accidents by sending an urgent message to producers or managers if greenhouse environmental data, which is collected from sensors in real time, corresponds to a some configured event value.

#### 3.2.2. Middleware Scenario

**Figure 6.**
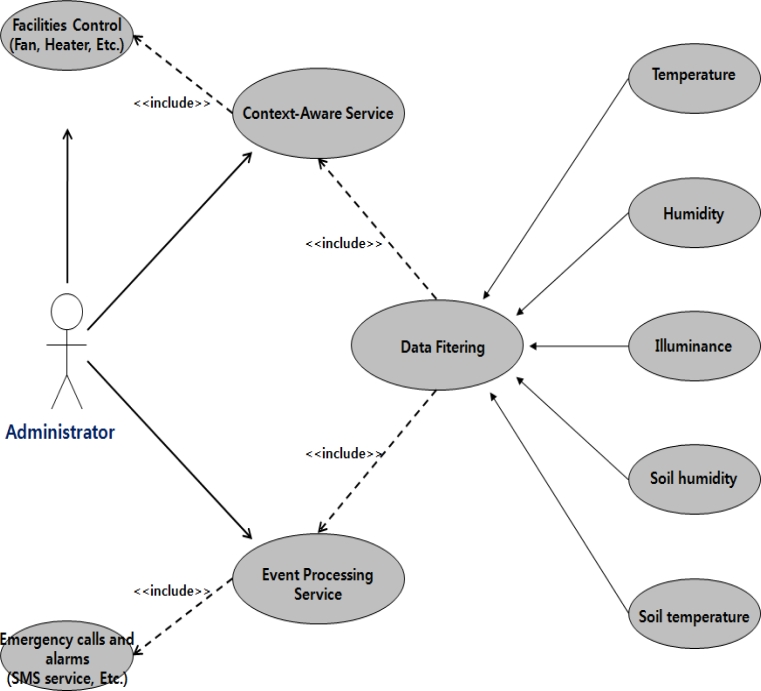
Use case diagram of the ubiquitous greenhouse system.

Figure 6 represents the functional demand analysis of the middleware system as a use case diagram. Producers or managers use the two context aware service and event processing service functions, and can control the greenhouse control facilities automatically or manually.

**Figure 7.**
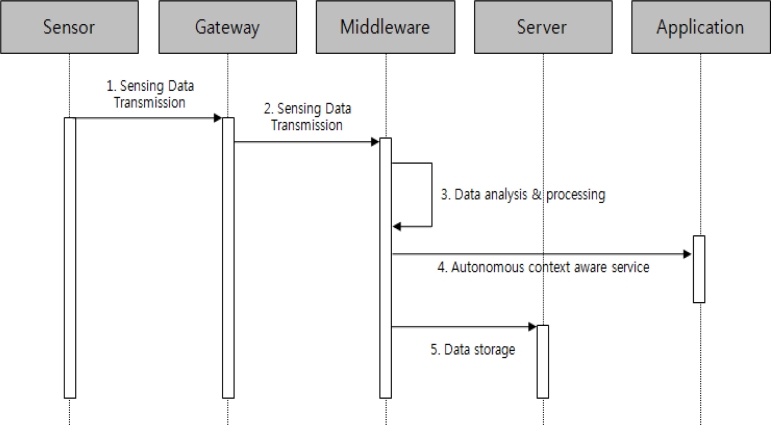
Sequence diagram of context aware service.

Figure 7 shows the process whereby the context aware service is carried out. If sensor data is entered from sensors, this data is sent to the middleware to perform the analysis and filtering process, and the autonomous context aware service is performed if it corresponds to a configured value of the context aware service.

**Figure 8.**
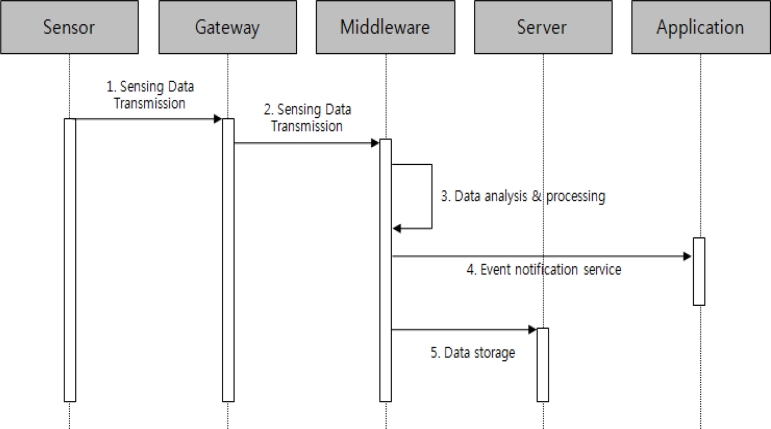
Sequence diagram of event service.

Figure 8 shows the process whereby the event service is carried out. Like the context aware service, it analyzes and processes data, which is sent to the middleware, to perform the event service if the data corresponds to a configured value of the event service.

**Figure 9.**
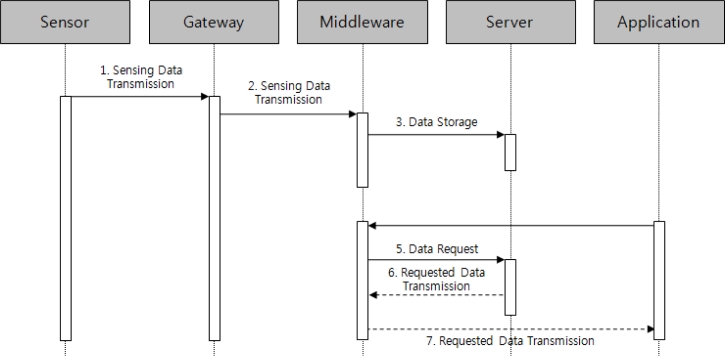
Sequence diagram of the greenhouse information search service.

Figure 9 represents a process that requests greenhouse data and image information stored in the database (DB), and the requested data is received. If the application manager accesses the middleware to request greenhouse information, the middleware requests the greenhouse information stored in the DB to be sent, and sends the received information to the application to make the greenhouse information monitoring possible.

#### 3.2.3. Middleware Design

**Figure 10.**
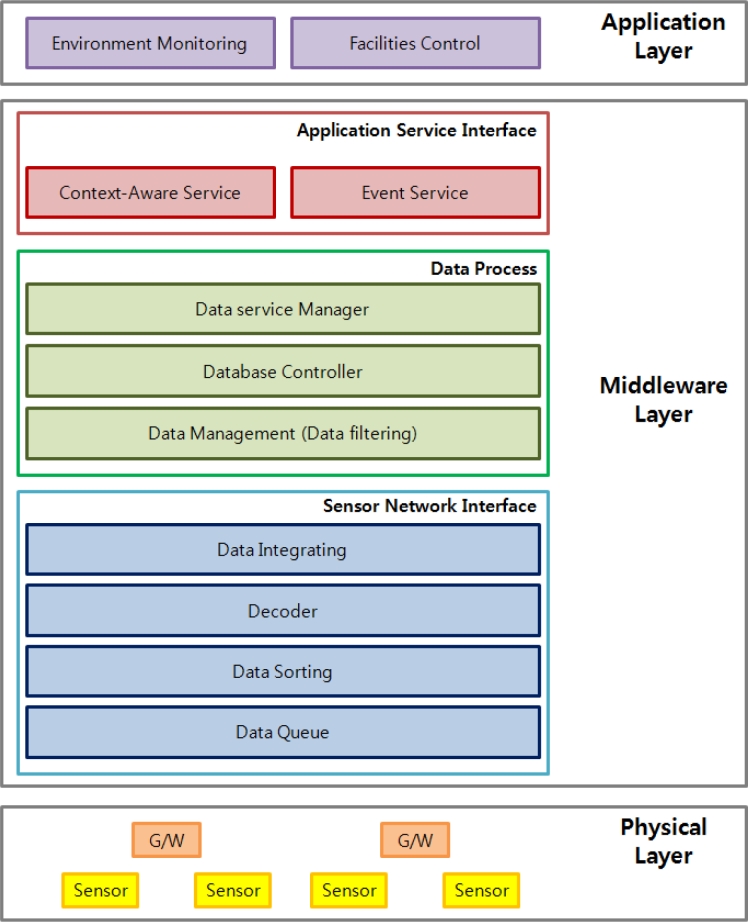
Hierarchical diagram of the proposed middleware.

The context aware middleware for u-Greenhouses is composed of a sensor network interface layer, data process layer, and application service layer, and Figure 10 shows an hierarchical diagram of the proposed middleware.

The sensor network interface layer is aimed at providing common interface functions to various multiple heterogeneous sensor networks, continuous monitoring and control functions for the states of various sensor networks.

The data process layer plays the role of providing various query processing functions for sensor data collected from the WSN infra and real-time management functions of sensor information. In addition, a sensor data management component is placed in the data processing layer to filter sensing data and support various forms of queries.

The application service interface layer, which is the top layer of middleware, plays the role of providing context aware and event processing services, and supports connections with the outside through query processing.

**Figure 11.**
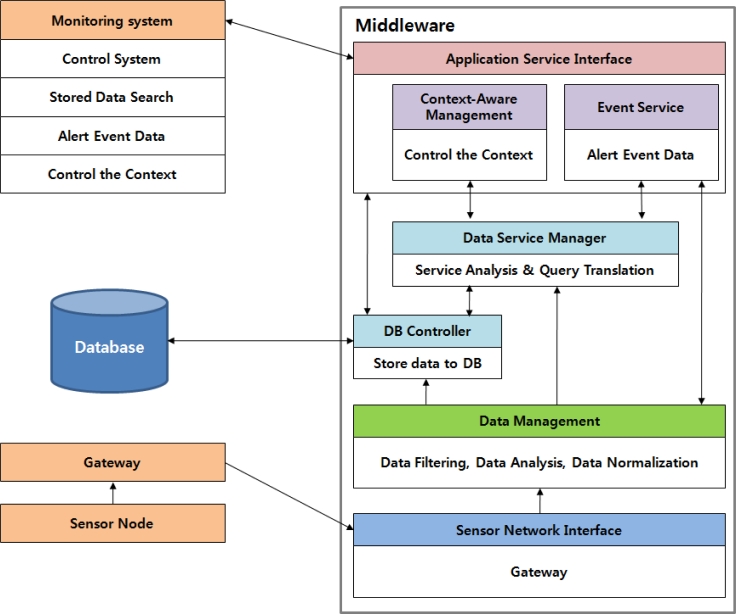
Structure and data flow chart of the proposed middleware.

Figure 11 represents the structure and data flow chart of the proposed middleware. Figure 11 shows functions for each module of the middleware and a structural flow with the sensors, gateway, and monitoring system.

The sensor network interface stores the different data forms of the heterogeneous sensor nodes in advance to make sure all the data ported in the heterogeneous sensor network environment can be recognized. The most ideal situation is to have an integrated transducer to convert into a common form all the data obtained from any heterogeneous sensor, however, since a standard for sensor node’s data forms is not established yet, this paper describes the conversion of two forms of data in the narrow concept into a specified common form.

**Figure 12.**
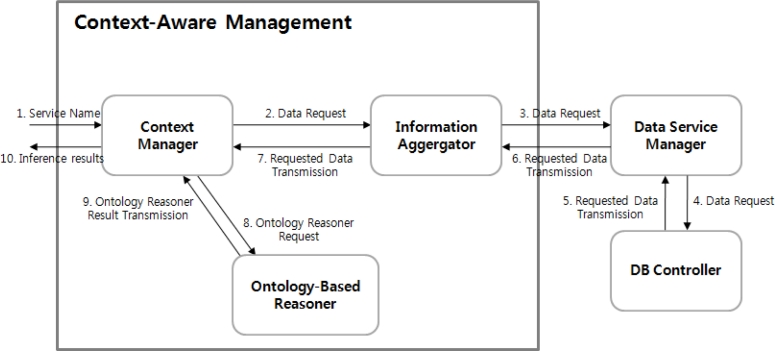
Structure and working process of the context aware management module

The data filtering does not store data delivered from the sensor network interface in the DB if the data is a duplicate, or stores data only when the data is changed. In addition, it makes context awareness possible by comparing with the data value configured in advance by the application to notify the context aware management module if it is identical or exceeds some threshold valued. Figure 12 shows the structure and working process of the context aware management module.

In addition, if the data entered has the same value as the level requested by an application, it notifies the event processing service module to make the event service possible. The usual sensing data not corresponding to such a process is delivered to the DB controller for storage in the DB.

The context-aware management module carries out the autonomous context-aware service to control the environmental control facilities in the greenhouse such as maintaining an optimum growth environment for the crops in the greenhouse depending on the data values based on data delivered from the data filtering. *Action_Value* and *Data_Value* are stored in the DB after the context-aware service.

The event service provides event services demanded by applications based on filtered data. It is a service to perform events specified by an application when receiving data corresponding to certain range of data values requested in advance. External intrusions and notifications of dangerous situations *etc.* correspond to this scenario. *Action_Value* and *Data_Value* are stored in the DB after the event service. The DB controller plays the role of sensing values delivered from the data filtering in the DB.

The application service interface plays the role of delivering and configuring a range of context aware services and event services configured by an application. And, it could search and store data in the database for items demanded by an application. In addition, it supports various queries to ensure flexible connections between applications and hardware are achieved.

## 4. Implementation of the Proposed Context-Aware Middleware for an Ubiquitous Greenhouse

### 4.1. Implementation Environment

The environment to implement the context-aware u-Greenhouse middleware is divided into hardware and software environments, and the details are as follows: the hardware environment to develop the proposed context-aware middleware is the server-side PC development environment, and the details are shown in Table 1.

In the hardware environment, the PC development environment corresponding to a server is implemented as the general PC environment, and the sensor node environment in charge of sensing is constructed as the environment suitable to use Zigbee sensors.

The software environment to develop the proposed context-aware middleware is as outlined in Table 2. In the software environment, the PC development environment uses a Microsoft Windows series operating system, Java and C# as programming language the and database is implemented with MySQL. The sensor node environment responsible for sensing is constructed as a Linux environment in the Windows environment with Cygwin, and it uses TinyOS as the operating system to produce the sensor program with NesC.

### 4.2. Implementation of the Proposed Context-Aware Middleware

#### 4.2.1. Middleware Algorithms

**Figure 13.**
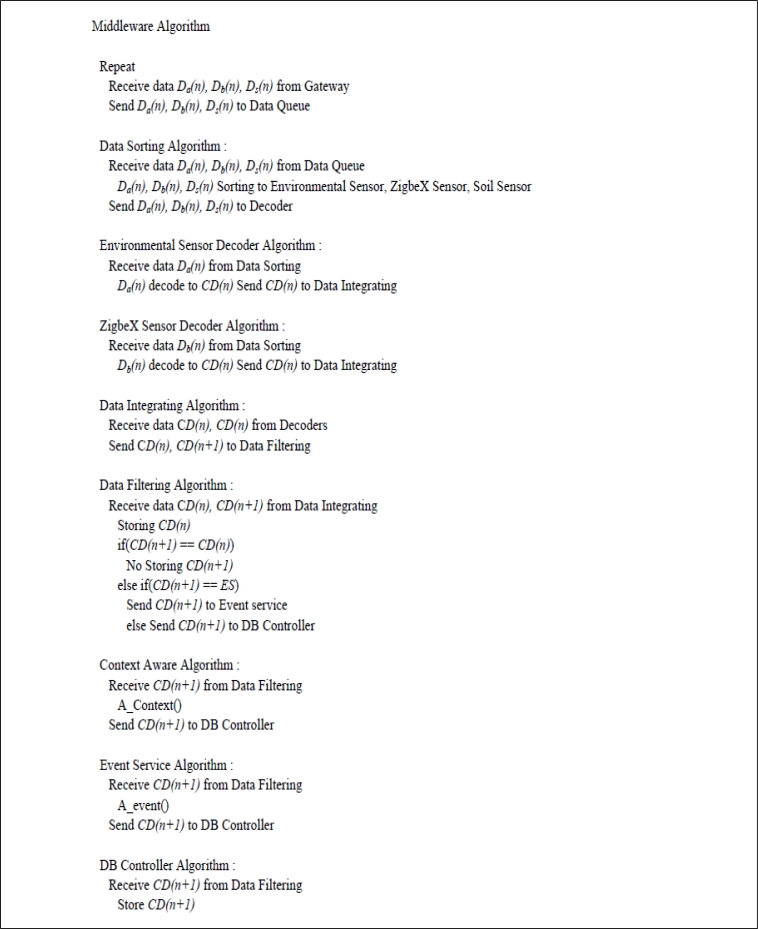
Middleware Algorithm.

Figure 13 is the algorithm that processes data which is delivered from the gateway in the implemented middleware, to store it in the DB.

Data of *Da(n)*, *Db(n)*, and *Ds(n)* obtained from the heterogeneous sensor networks are divided into each form in the data sorting module to send to each decoder. Each decoder converts *Da(n)*, *Db(n)*, and *Ds(n)* into the common form *CD(n)*, and sends to the data integrating module to integrate it as *CD(n)* and *CD(n + 1)* in order.

Data integrated as the common form is not sent to the DB controller module and dropped if it is duplicated data by comparing *CD(n)* and *CD(n + 1)* continuously entered through the data filtering module.

In addition, when data identical to the data range configured in advance by the context-aware and event service module is sent, it goes to the context-aware and event service, respectively, to make them perform the corresponding action. The remaining values are sent to the DB controller module to store in the DB.

#### 4.2.2. Sensor Data Structure and Data Conversion

The data obtained from heterogeneous sensor networks with sensors measuring illuminance, temperature, humidity, EC, pH, soil humidity, *etc.* is converted into a common form for use. This is possible by converting the data structure in the packet units into the specified common form.

**Figure 14.**
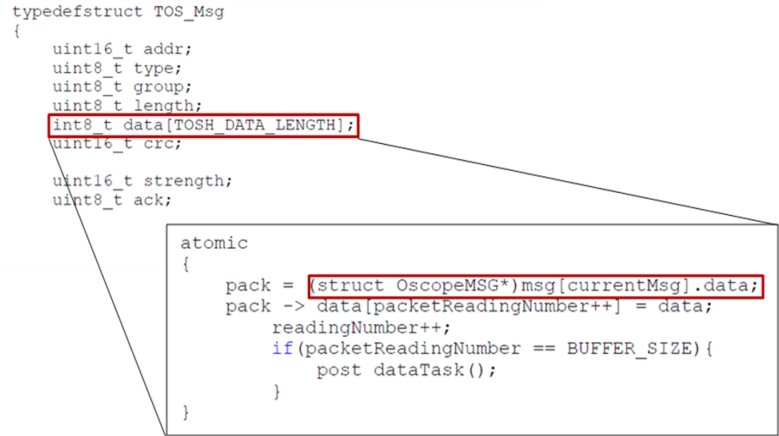
Sending message structure and data conversion.

Figure 14 shows the structure of the message (TOS_Msg msg[],) which sends data defined already for abstraction converting into the common form, and data structure int8_t data[TOSH_DATA_LENGTH] containing the corresponding data.

#### 4.2.3. Implementation of Ontology for Context-Aware Service

**Figure 15.**
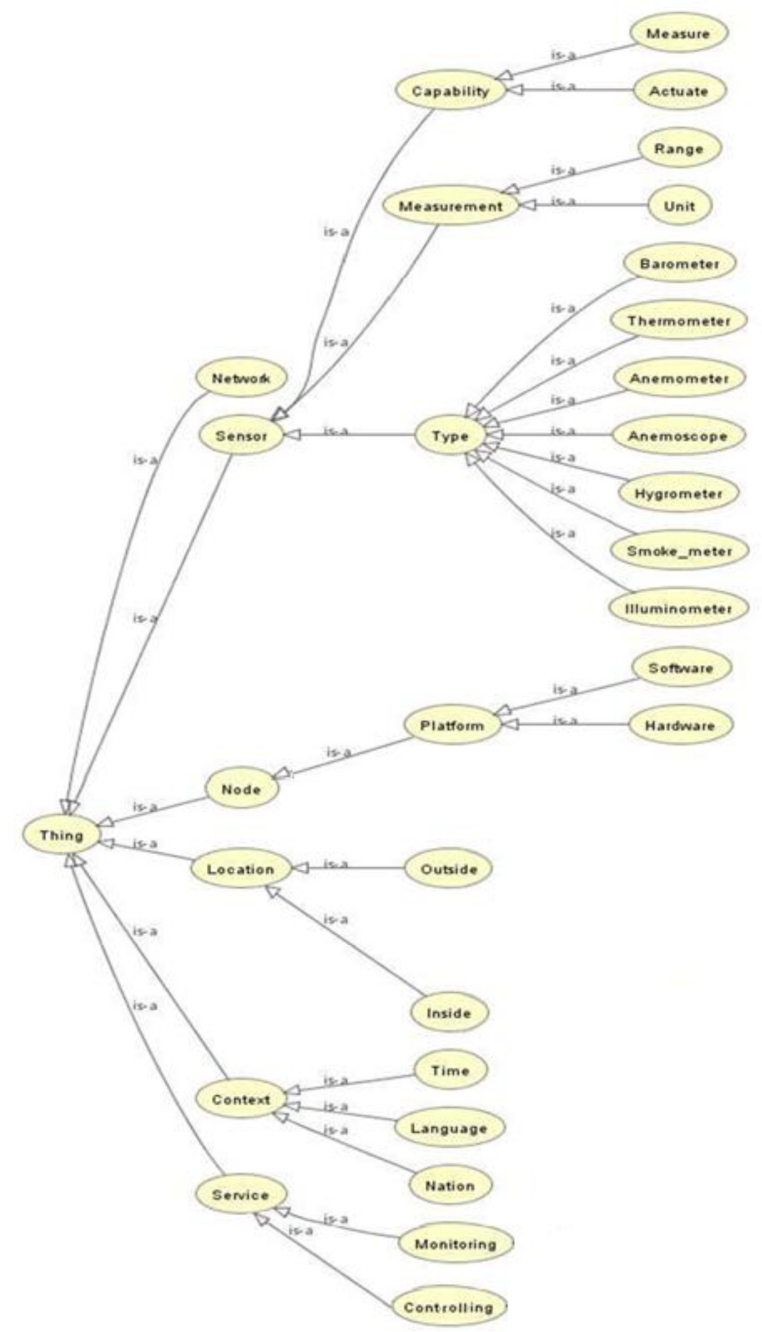
Class hierarchies of ontology.

To provide context-aware service, an ontology is constructed as shown in Figure 15; the ontology design uses Protege [33]. The ontology of this paper has six higher classes. Network represents information of sensor networks, and Sensor, Node, Location, and Context represent sensor, node, location, and context information, respectively, and Service represents services that users could be provided in the u-Greenhouse. Such information is written as OWL [34] documents and used with JENA [35].

### 4.3. Application of the Proposed Middleware

**Figure 16.**
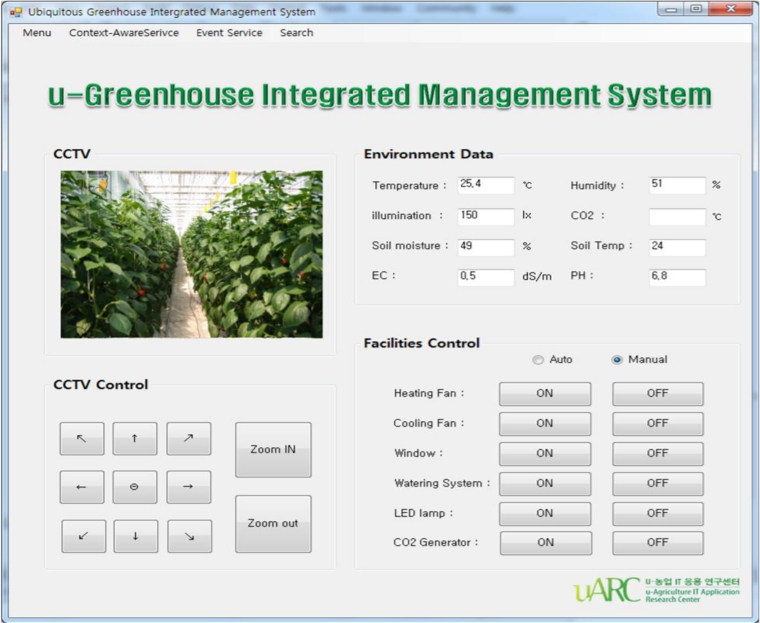
u-Greenhouse integrated management system GUI.

The proposed middleware in this paper was applied to an existing u-Greenhouse to implement the u-Greenhouse integrated management system. Figure 16 shows the UI of u-Greenhouse integrated management system applying the proposed middleware. The system UI displays information such as illuminance, temperature, humidity, CO_2_, *etc.* collected from the environmental and soil sensors installed in the greenhouse as a text form, enabling real-time image information of the greenhouse and also controlling the CCTV, and allowing notification of the working state of the greenhouse environmental control facilities and their control. In addition, it was implemented so as to allow managers to directly set a range of context-aware and event services so that it could provide context-aware serviced such as automatic control of the greenhouse environment, *etc.* and the SMS notification service for dangerous situations.

**Figure 17.**
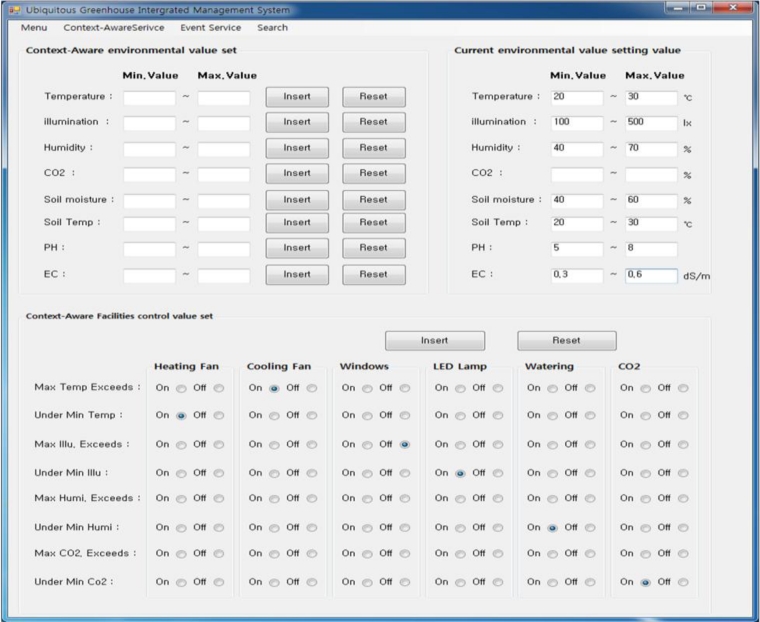
Context-aware service GUI.

**Figure 18.**
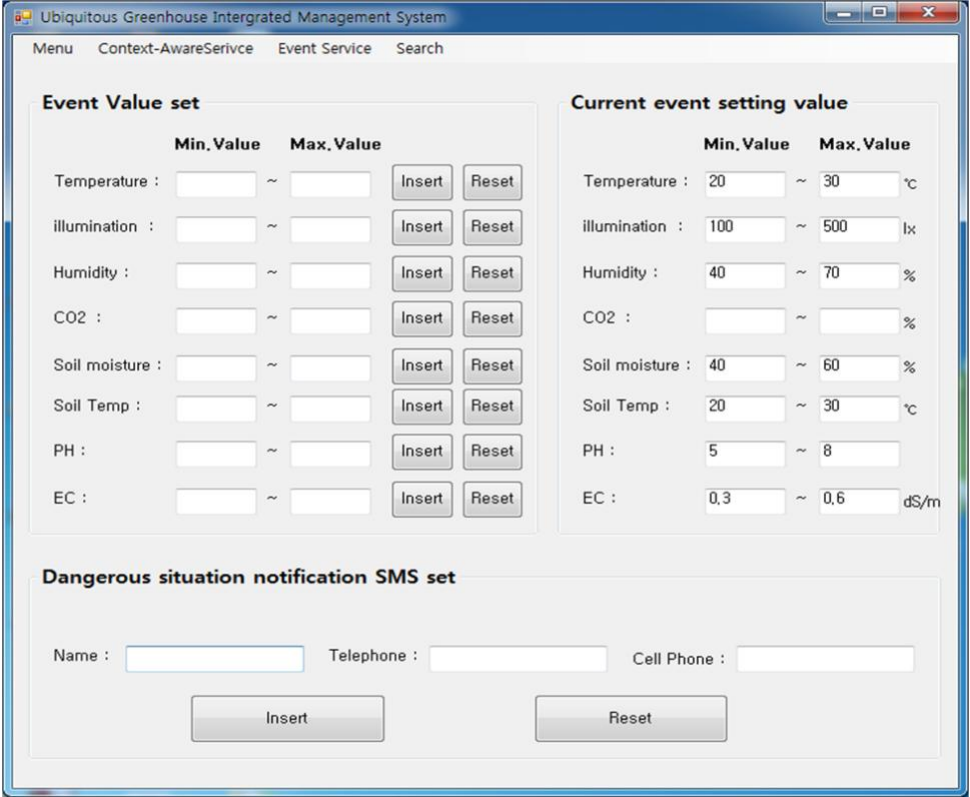
Event service GUI.

Figures 17 and 18 are screens that set a range of context-aware and event services.

### 4.4. Performance Evaluation of the Proposed Middleware

**Figure 19.**
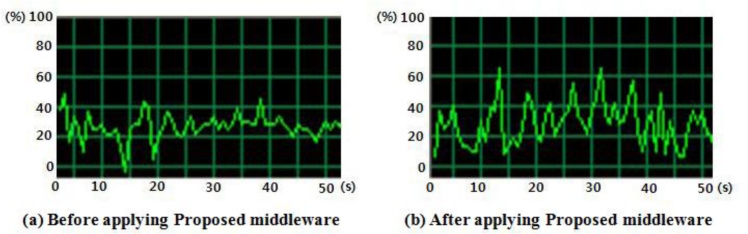
Usage of CPU.

This paper confirmed the proposed middleware’s operability by measuring the server’s load level and the response time for user's queries. To indirectly compare and measure the load level when integrating and processing data generated from lots of sensors, we used an application offered by Microsoft Windows. It is shown that CPU usage was about 20∼30% on average when processing sensing data without middleware, and about 30∼40% on average when processing data with the proposed middleware, as seen in Figure 19. Even though the value using the middleware is a little higher than the existing method processing data without middleware, it does not exceed 50% on average and considering that context-aware service, which it is not provided in the existing system, is added, therefore it could be confirmed that it is useful in terms of system usability.

**Figure 20.**
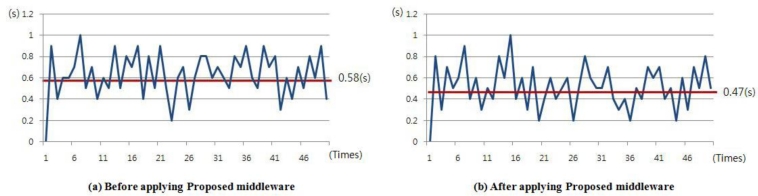
Average response time for user’s requests.

In addition, in order to measure average response time for user's requests the time required to obtain requested data from the server was simulated 50 times, and the result is as seen in Figure 20.

As seen in the figure, the response speed for user’s requests was 0.58 s on average before using the middleware, and after applying the proposed middleware the average response speed was 0.47 s, therefore, it could be seen that there was an improvement in terms of response speed.

## 5. Conclusions

This paper proposed and developed a context-aware middleware to efficiently process data collected from a u-Greenhouse applying WSN technologies and to implement combined services through organic connections of data. In order to solve the problem of dependence on certain hardware and operating systeme, which is a disadvantage of the existing middleware products, a novel middleware integrating different forms of data was suggested, and operability and scalability of the middleware was maximized by adding intelligent context-aware, event service, and filtering functions. In addition, to evaluate the performance of the proposed middleware, it was applied to an existing u-Greenhouse to implement a u-Greenhouse integrated management system, and it could be confirmed that it was useful in terms of usability by comparing the CPU usage load level with that of the existing system and the response time for user’s requests when operating the system. In future studies, the integrated middleware supporting abstraction between heterogeneous middleware for system’s scalability by applying the future Web service should be studied, and expansion of ontology is needed for more accurate intelligent services. If such studies are reflected in the future versions of the middleware, it is expected that utilization of middleware would be increased.

## Figures and Tables

**Table 1.** Hardware Environment.

	Type	Details

Server PC Environment	CPU	Intel Xeon 3.2 Ghz
	RAM	1 GB
	OS	Microsoft Windows XP

**Table 2.** Software Environment.

	Type	Details

PC Development Environment	OS	Microsoft Windows XP
	Programming Languages	JAVA (JDK 6), C#
	RDBMS	MySQL 5.0

Sensor Node Environment	OS	TinyOS 1.0
	Linux Environment	Cygwin
	Sensor Programming Languages	NesC
	JAVA	JDK 1.4.1
